# Issues in the 3rd year of the COVID-19 pandemic, including computer-based testing, study design, ChatGPT, journal metrics, and appreciation to reviewers

**DOI:** 10.3352/jeehp.2023.20.5

**Published:** 2023-01-31

**Authors:** Sun Huh

**Affiliations:** Department of Parasitology and Institute of Medical Education, College of Medicine, Hallym University, Chuncheon, Korea; Hallym University, Korea

## More active adoption of computer-based testing in health professional licensing examination in Korea

**Fig. 1.**
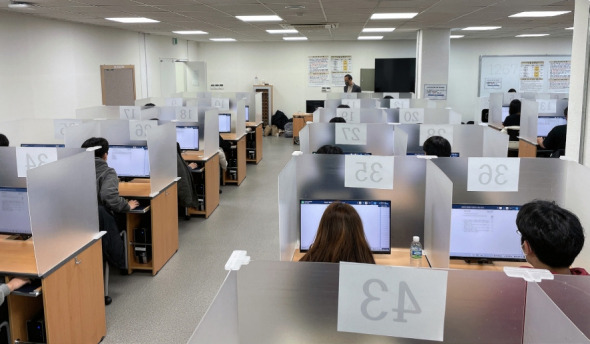
Photo of the permanent Wonju Testing Center where examinees took a Korean Medical Licensing Examination through computer-based testing on January 5, 2023 (Courtesy of the Korea Health Personnel Licensing Examination Institute).

In 2022, computer-based testing (CBT) was introduced for the Korean Medical Licensing Examination (KMLE). CBT has also been expanded to the Korean Dental Licensing Examination, Korean Oriental Doctor Medical Licensing Examination, and Korean Care Worker Licensing Examination in 2023. Subsequently, 26 licensing examinations will be administered through CBT in 2025 [1]. For a more convenient and stable testing environment, the Korea Health Personnel Licensing Institute prepared 9 permanent test centers for CBT, which collectively have 1,500 seats (Fig. 1). If the number of examinees on a particular date surpasses 1,500, other sites will be leased.

No technical problems occurred during the implementation of CBT for the KMLE. All medical schools in Korea adopted CBT, and no examinee complained of any difficulties in taking CBT. However, further improvements should be made after the transition to CBT.

First, the standard setting of CBT still has not been adopted, although there were some studies on the cut score in the 2022 volume of the *Journal of Educational Evaluation for Health Professions* (JEEHP). Kim et al. [2] suggested that acceptable cut scores for the KMLE could be derived and proposed independently using the yes/no Angoff and Hostee methods. Park [3] recommended using the yes/no Angoff method with a 60% probability for deciding “yes” as a substitute for the percent Angoff method in estimating the cutoff score of the KMLE. According to this theoretical background, it is time to consider standard setting for health professions licensing examinations instead of using a 60% correct answer rate as the threshold for passing.

Second, more variable item types are recommended to take advantage of CBT’s strengths: drawing charts, drag-out & in, rich media scenarios, ordered responses, and hotspots. In particular, more multimedia test items are recommended to reflect the actual clinical situation. In the 2022 KMLE, 3 items included multimedia components, and 6 items will do so in 2023.

## How to deal with manuscripts or paragraphs generated by ChatGPT

After the appearance of ChatGPT―an artificial intelligence chatbot in November 2022, there may be submissions where the authors have received help from this chatbot, especially in the Introduction section. There is a debate on the authorship of ChatGPT [4]. However, JEEHP will not consider to accept ChatGPT as an author, but ChatGPT-generated sentences or paragraphs may be cited. It is nearly impossible to detect whether ChatGPT generates text, because paragraphs generated using ChatGPT usually cannot be screened by plagiarism detection programs, including Similarity Check. Sources do not accompany ChatGPT-generated content. Therefore, it is recommended to verify the sources generated by ChatGPT for citation in the main text. Authors should meticulously check whether ChatGPT-generated content is accurate and suitable, because answers generated by ChatGPT are not perfect in response to questions about specific fields, such as parasitology [5] or digital standards of journal publishing [6].

## Country name, study design, and corresponding reporting guidelines to improve the scientific quality

I have asked the authors of manuscripts submitted to JEEHP to specify the name of the country of the study population in the title since 2015. This information is included to stimulate readers’ interest in JEEHP articles. Marking country names has been frequently required in clinical studies. Knipe and Jewkes [7] also recommended that authors include the country name in which human studies were conducted in the report title. They said, “All research is a product of the social and cultural system within which it is created, and therefore, it should be labeled.” I agree with them. Although there is still no evidence that the country name has attracted much attention, I believe this information can elicit interest among researchers in the corresponding country.

Kim [8] reviewed the study design and corresponding reporting guidelines and stated that out of 44 articles published in JEEHP from January 2021 to September 2022, 19 (43.2%) needed a more suitable study design, particularly pointing out this issue for before-and-after studies, diagnostic research, and non-randomized trials. The editorial office will be more cautious in suggesting an appropriate study design to the authors. Furthermore, we will continue emphasizing reporting guidelines for the manuscript description. This reporting style may help readers easily identify interesting parts of an article.

## Journal metrics and statistics

The 2nd Journal Citation Indicator (JCI) value announced in June 2022, 0.58, showed an improvement from 0.51 in 2021. This JCI ranking in the scientific education category was 10th out of 38 Emerging Sources Citation Index (ESCI) journals (73.7%) and 46th out of 82 Science Citation Index Expanded (SCIE) and ESCI journals (43.9%). This journal’s performance in terms of citations was similar to that in 2021 [9].

**Fig. 2.**
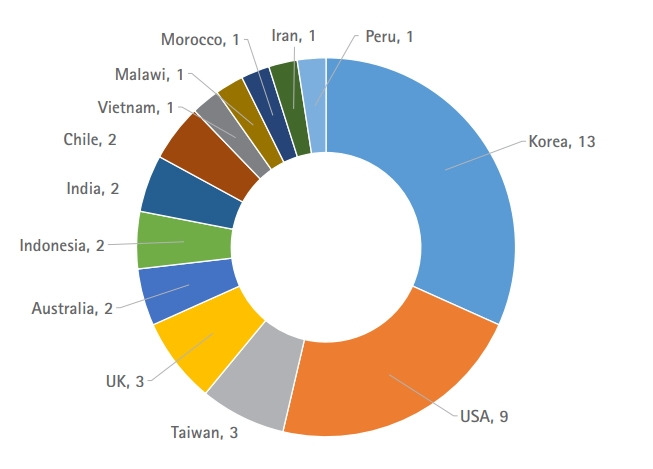
Number of articles in the *Journal of Educational Evaluation for Health Professions* according to the authors’ country in 2022.

The authors’ countries in the 2022 issue are shown in Fig. 2. The total cites in Crossref metadata, Scopus, and Web of Science Core Collection in 2022 were estimated as 825, 845, and 666 times, respectively, on January 23, 2023, reflecting a substantial increase from the values of 525, 555, and 461, respectively, in 2021 [9]. CiteScoreTracker 2022, provided by Elsevier B. V., is 4.3 (650 citations to date/151 documents to date) on January 23, 2023 (https://www.scopus.com/sourceid/21100467423). The 2022 Journal Impact Factor (JIF), which will be officially announced in June 2023, is estimated as 4.286 (300 cites in 2022 for articles in 2020–2021/70 citable articles in 2020–2021). The JIF ranking is estimated as the 7th out of 82 in the “scientific discipline education” category. In the previous editorial, the 2021 JIF was estimated as 1.846, but the final manually calculated value was 2.103 [9]. Journal statistics in 2022 are presented in Table 1.

The editorial office tried not to reject peer-reviewed manuscripts to save the reviewers’ time. Unfortunately, out of 56 reviewed manuscripts, 10 were rejected and 4 were withdrawn by the authors. With the exclusion of 5 manuscripts in revision, the acceptance rate of peer-reviewed unsolicited manuscripts was 70.8% (34/48), which is almost the same as the rate in 2021 (70.3%, 26/37). In 2023 and beyond, the editorial office will do its best to increase the acceptance rate of unsolicited manuscripts to at least 80% if they enter the peer review process. On October 20, 2022, *eLife* announced that it would no longer act as a gatekeeper and would publish all peer-reviewed manuscripts along with the reviewers’ feedback. This change means that readers can evaluate the worth of each article by reading both the manuscript and the comments from the reviewers [10]. JEEHP still has not accepted this open peer-review policy, but the goal is the same as that of *eLife*―not to waste reviewers’ valuable comments and to let readers determine the value of the manuscripts. We will try to accept any manuscripts meet the aims and scope and fit the style and format of the journal. Of course, budgetary limitations remain, and it is challenging to publish more than 40 articles a year. The editorial office will also try its best to shorten the median time until the first decision to 14 days at most.

## Reviewers and volunteers’ devotion to journal publishing

In 2022, many reviewers voluntarily participated in peer review. I appreciate that they shared their time to enhance the quality of articles published in the journal. The following list presents the reviewers and their affiliations in 18 countries:

**Australia:** Wayne C. Hodgson, Monash University; Boaz Shulruf, The University of New South Wales

**Canada:** Sarah Ae Aboushawareb, McGill University; Rashid Coomal, University of Toronto; Marguerite Roy, Medical Council of Canada; Harman Singh Sandhu, University of Toronto; Timothy J. Wood, University of Ottawa

**Chile:** Gabriel Andrade, University of the Andes; Manuel Castillo Niño, Universidad Mayor; Silvana Castillo Parra, Universidad de Chile; Daniela Pino Valenzuela, University of Concepcion

**Czech Republic:** Alan Mejstrik, Emergency Medical Service, Prague

**Indonesia:** Sigit Purbadi, University of Indonesia; Tofan Widya Utami, Universitas Indonesia

**Iran:** Abdolghani Abdollahimohammad, Zabol University of Medical Sciences; Maryam Alizadeh, Tehran University of Medical Sciences; Fateme Jafaraghaee, Guilan University of Medical Sciences; Nazila Javadi, Guilan University of Medical Sciences; Samad Karkhah, Guilan University of Medical Sciences; Mohammadreza Mobayen, Guilan University of Medical Sciences; Ali Norouzi, Zanjan University of Medical Sciences; Siamak Rimaz, Guilan University of Medical Sciences; Amir Emami Zeydi, Mazandaran University of Medical Sciences

**Japan:** Fitriana Nur Rahmawati, Osaka University

**Jordan:** Ashraf Khasawneh, Hasheimte University

**Korea:** Duck-Sun Ahn, Korea University; Su Jin Chae, Ajou University; A Ra Cho, Catholic University of Korea; Eun Kyung Choi, Kyungbuk National University; Eun Young Choi, Sungshin Women’s University; Yun-Kyung Choi, Korea National Open University; Sunmi Han, Sookmyung Women’s University; Yera Hur, Hallym University; Geum Hee Jeong, Hallym University; Chul-Gyu Kim, Chungbuk National University; Jihyun Kim, Ewha Women’s University; Jisu Kim, Chung-Ang University; Jooah Kim, Yonsei l University; Sun Kim, Catholic University of Korea; Young-Min Kim, Catholic University of Korea; Eunkyung Lee, Kyung-in Women’s University; Seung-Hee Lee, Seoul National University; Jakyung Min, Samsung Medical Center; Younjae Oh, Hallym University; Janghee Park, Soonchunhyang University; Jungchul Park, Yonsei University; Song Yi Park, Dong-A University; Suyeon Park, Soonchunhyang University; Ji-Hyun Seo, Gyeongsang National University; Dong Gi Seo, Hallym University; Sujin Shin, Ewha Women’s University; Yeonok Suh, Soonchunhyang University; Mi Kyoung Yim, Korea Health Personnel Licensing Examination Institute; Hyun-Sun Yoon, Seoul National University Boramae Medical Center

**Morocco:** Hasnaa Sine, Mohammed V University of Rabat; Rachid Racine, Mohammed V University of Rabat

**New Zealand:** Marcus A. Henning, University of Auckland; Poole Phillippa, University of Auckland; Sarah Randa, Universities New Zealand - Te Pōkai Tara; Tim J Wilkinson, University of Otago

**Peru:** Niels Pacheco, Universidad Peruana Cayetano Heredia; Franklin Peralta, Usamedic scrl

**Spain:** Juan-José Igartua Perosanz, University of Salamanca

**Taiwan:** Fen-Yu Tsen, National Taiwan University Hospital

**Thailand:** Pallop Siewchaisakul, Chiang Mai University

**Turkey:** Fezile Özdamli, Near East University

**United Kingdom:** Fiona Myint, University College London; Cesar A. Orsini, University of East Anglia

**United States:** Brad Allen, Texas Tech University Health Sciences; Jean M. Bailey, Virginia Commonwealth University; Jean-Michel Brismée, Texas Tech University Health Sciences Center; Jennifer Dice, Texas Children’s Hospital; Chasity Falls, University of Michigan-Flint; Collen Jacob, Uniformed Services University; Dice Jennifer, Texas Children’s Hospital; Kelvin Kenyoru, University of Texas at Tyler; Andrew Krouse, University of Texas at Tyler; Katherine Myers, Duke University; Thong Ba Nguyen, University of Hawaii at Manoa; Erika Ozdemirer, Gaylord Rehabilitation Hospital; Patrice Paolella, Rutgers University; Andrea Pelletier, Harvard Medical School; Kelly Braden Reynold, Duke University; Leigh Ronald, Carolton Chronic and Convalescent Hospital; Suzanne Rose, Stamford Health; Katherine Stone, University of Texas at Tyler; Gabriel R. Sudario, University of California Irvine; Jonathan Webb, Mayo Clinic; Michelle Wormley, Sacred Heart University; Hon Yuen, University of Alabama at Birmingham

Tom Huh, a graduate student in the Division of Life Sciences, College of Life Sciences and Biotechnology, Korea University, Seoul, Korea, recorded some audio abstracts voluntarily.

## New year’s plan for the journal publishing

We are ready to introduce graphic abstracts for manuscripts submitted in 2023. A professional designer will complete the graphic abstract when authors draw the article content as a figure. This graphic abstract will attract more attention from readers and be able to be used for conference presentations and for education in the classroom.

The reference style for the internet website will be changed from 2023. The present style is as follows: [Author]. [Title] [Internet]. [Place of publication]: [Publisher]; [Date of publication] [Date of update/revision; Date of citation]. [Availability]

The example of the present style is as follows:

National Health Licensing Examination Board. Clinical skill test [Internet]. Seoul (KR): National Health Licensing Examination Board; 2020 [cited 2023 Jan 30]. Available from: https://www.kuksiwon.or.kr/EngHome/cnt/c_3109/view.do?seq=18

From 2023, it will be changed as follows: first, Place of publication is deleted; second, Date of publication can be omitted if no date is provided; third, Author, Publisher, or Date of update/revision also can be omitted if it is not indicated. Therefore, the minimum requirement of website citation is as follows: [Title] [Internet]. [Date of citation]. [Availability]

The example is as follows:

Clinical skill test [Internet]. [cited 2023 Jan 30]. Available from: https://www.kuksiwon.or.kr/EngHome/cnt/c_3109/view.do?seq=18

In 2022, there were 3 articles on licensing examinations. JEEHP’s primary scope encompasses health professions licensing examinations. Therefore, the editorial office will try to receive or invite those manuscripts more actively. It is essential to collaborate with other countries’ institutes that have dealt with national licensing examinations for health professions. I welcome suggestions from those international institutions for collaboration for publishing their works.

It is the 19th year that I have served as the editor-in-chief of JEEHP. I would not have expected in 2005 that I would voluntarily devote myself to JEEHP for such a long time. It has genuinely been my pleasure to edit these manuscripts for authors and readers, and I am happy to continue doing this work during my editorship.

## Figures and Tables

**Table 1.** Journal statistics of manuscripts submitted to the *Journal of Educational Evaluation for Health Professions* from January 1 to December 31, 2022

	No.	Content
Manuscripts submitted	281	
No. of commissioned manuscripts	7	Editorial, 4; review, 1; research article, 1; educational/faculty development materials, 1
No. of unsolicited manuscripts	274	
Manuscripts rejected without peer-review	221	Unsuitable, 211; other reasons, 10
Manuscripts peer-reviewed out of 274 unsolicited manuscripts	53	Published, 31; accepted and under editing, 3; rejected, 10; withdrawn, 4; in revision, 5
No. of publications out of 281 submitted manuscripts	41	
Acceptance rate overall (%)	14.6	41/281=0.146
Acceptance rate of unsolicited manuscripts (%)	12.4	34/274=0.124
Median time from submission to the first decision (days)	20	
Median time from submission to publication (days)	68	
Median time from acceptance to publication (days)	1	
